# Association between vestibulo-ocular reflex suppression, balance, gait, and fall risk in ageing and neurodegenerative disease: protocol of a one-year prospective follow-up study

**DOI:** 10.1186/s12883-015-0447-5

**Published:** 2015-10-09

**Authors:** Karin Srulijes, David J. Mack, Jochen Klenk, Lars Schwickert, Espen A. F. Ihlen, Michael Schwenk, Ulrich Lindemann, Miriam Meyer, Srijana K.C., Markus A. Hobert, Kathrin Brockmann, Isabel Wurster, Jörn K. Pomper, Matthis Synofzik, Erich Schneider, Uwe Ilg, Daniela Berg, Walter Maetzler, Clemens Becker

**Affiliations:** Department of Neurodegeneration, Hertie Institute for Clinical Brain Research, University of Tuebingen, Tuebingen, Germany; Department of Geriatrics and Clinic of Geriatric Rehabilitation, Robert-Bosch-Hospital, Stuttgart, Germany; Department of Cognitive Neurology, Hertie Institute for Clinical Brain Research, University of Tuebingen, Tuebingen, Germany; Clinic for Neurology, University Hospital Zurich, Zurich, Switzerland; Institute of Epidemiology and Medical Biometry, Ulm University, Ulm, Germany; Department of Neuroscience, Norwegian University of Science and Technology, Trondheim, Norway; German Research Center for Neurodegenerative Diseases (DZNE), University of Tuebingen, Tuebingen, Germany; Institute of Medical Technology, Brandenburg University of Technology Cottbus –Senftenberg, Cottbus, Germany

**Keywords:** Vestibulo-ocular reflex suppression, VOR, Falls, Postural stability, Gait, Balance, Neurodegeneration, Parkinson’s Disease, Parkinsonism, Older persons

## Abstract

**Background:**

Falls frequency increases with age and particularly in neurogeriatric cohorts. The interplay between eye movements and locomotion may contribute substantially to the occurrence of falls, but is hardly investigated. This paper provides an overview of current approaches to simultaneously measure eye and body movements, particularly for analyzing the association of vestibulo-ocular reflex (VOR) suppression, postural deficits and falls in neurogeriatric risk cohorts. Moreover, VOR suppression is measured during head-fixed target presentation and during gaze shifting while postural control is challenged. Using these approaches, we aim at identifying quantitative parameters of eye-head-coordination during postural balance and gait, as indicators of fall risk.

**Methods/Design:**

Patients with Progressive Supranuclear Palsy (PSP) or Parkinson’s disease (PD), age- and sex-matched healthy older adults, and a cohort of young healthy adults will be recruited. Baseline assessment will include a detailed clinical assessment, covering medical history, neurological examination, disease specific clinical rating scales, falls-related self-efficacy, activities of daily living, neuro-psychological screening, assessment of mobility function and a questionnaire for retrospective falls. Moreover, participants will simultaneously perform eye and head movements (fixating a head-fixed target vs. shifting gaze to light emitting diodes in order to quantify vestibulo-ocular reflex suppression ability) under different conditions (sitting, standing, or walking). An eye/head tracker synchronized with a 3-D motion analysis system will be used to quantify parameters related to eye-head-coordination, postural balance, and gait. Established outcome parameters related to VOR suppression ability (e.g., gain, saccadic reaction time, frequency of saccades) and motor related fall risk (e.g., step-time variability, postural sway) will be calculated. Falls will be assessed prospectively over 12 months via protocols and monthly telephone interviews.

**Discussion:**

This study protocol describes an experimental setup allowing the analysis of simultaneously assessed eye, head and body movements. Results will improve our understanding of the influence of the interplay between eye, head and body movements on falls in geriatric high-risk cohorts.

**Electronic supplementary material:**

The online version of this article (doi:10.1186/s12883-015-0447-5) contains supplementary material, which is available to authorized users.

## Background

Worldwide, the number of persons aged 60 and older is expected to almost triple from 841 million in 2013 to over two billion in 2050 [1]. Since falls are more common [2] and their consequences often more severe [3] in the older population, this demographic development will be accompanied by an increased frequency of falls. The according personal, social and economic burden will have a strong impact on society. Many risk factors for falls like gait and balance deficits [2] have been identified in the older population, but the contribution of failures in the interplay between oculo- and locomotion (especially combined head and body movement) to the risk of falls has not yet been studied in detail. This is surprising given that visual exploration of the environment while standing or walking is regularly required during daily life (e.g. crossing a street), and deficits in the oculomotor system may have impacts on the performance of the locomotor system, and vice versa. Hollands and Marple-Horvat provided evidence for the interaction of the oculo- and locomotor systems during a visually guided stepping task [4]. They found that any variation in stance duration was accompanied by a variation in timing of saccade onset with the interval between saccade onset and foot lift being constant. Glasauer and colleagues described another interaction between eye movements and postural control: not only retinal slip provides a relevant feedback for postural control, but also slow eye movements [5].

Importantly, different tasks require different oculomotor strategies. During normal ambulation and head movement, the vestibulo-ocular reflex (VOR) stabilizes gaze, and therefore helps to keep the image of the environment stable on the retina. This reflex enables *antagonistic* eye and head movements. In contrast, maintaining gaze on a moving object or shifting gaze to a new object of interest e.g., while walking on the sidewalk and suddenly fixating a car driving towards oneself, requires the eyes and the head to move into the same direction. For the proper performance of these *agonistic* concerted movements, the suppression of the vestibulo-ocular reflex (VOR suppression) is inevitable [6, 7]. The VOR suppression enables the guidance of the eyes into the direction of the head during self-generated directional head movements, and can be performed either very fast (while gaze shifting, see examples above) or smoothly (e.g., when walking and pursuing a car driving past). During natural visual exploration in ecologically valid environments 22 % of eye movements do not compensate for the head movements, but are synergistic [8].

Two different forms of eye movements are used to change gaze, which is to move the eye in space to follow a moving target – smooth pursuit; or to redirect the eye to a new target of interest – saccades. As a consequence, the image of the target is presented on the fovea (area of highest resolution) in both cases. Saccades are very fast gaze shifts which rapidly place an object of interest onto the fovea, whereas smooth pursuit is slow. Head movements can be added to both forms of eye movements to achieve a wider range of motion [9]. The underlying neuronal systems are different for fast eye/head movements and smooth eye/head movements which illustrate the need to analyze VOR suppression separately for both types of gaze changes.

### Vestibulo-ocular reflex suppression during smooth eye-head pursuit

Two separate mechanisms have been discussed to enable VOR suppression during eye-head pursuit, that is to enable to overcome the vestibular drive that would move the eyes away from the target of interest. The first is the cancellation of the VOR by a smooth-pursuit signal based on a cerebro-ponto-cerebellar pathway [9]. The second mechanism is a partial, parametric reduction of VOR gain (eye velocity/head velocity) [10].

Three types of neurons in the vestibular nuclei are probably involved in both processes: position-vestibular-pause neurons, vestibular-only neurons and flocculus target or eye-head neurons [10]. Concerning the first mechanism, eye-head neurons seem to modulate their discharge during smooth pursuit and eye-head tracking, by possibly receiving a pursuit signal from the vestibulocerebellum. The second mechanism of reduction of VOR gain seems to be achieved by the attenuation of the discharge of position-vestibular-pause neurons. This mechanism might be elicited by matching of proprioceptive signals from the neck and the according, expected signals of the neck motor command [11]. These pathways point to the complexity of regulation of the VOR suppression, involving a large network of brain areas and, therefore, being vulnerable to the risk of age- and brain lesion-associated deficits of this movement.

### Vestibulo-ocular reflex suppression during rapid eye-head gaze shifts

Looking down on the pavement to step over a hole is made possible by a highly coordinated fast eye-head movement. To perform such larger eye-head gaze shifts beyond the oculomotor range, saccadic and vestibular signals have to be well coordinated.

A saccade is a rapid eye movement that shifts the line of sight between points of fixation [10]. Controlling circuits involve the superior colliculus, the frontal and parietal eye field and the horizontal and vertical brainstem saccade generators [12].

**Fig. 1:**
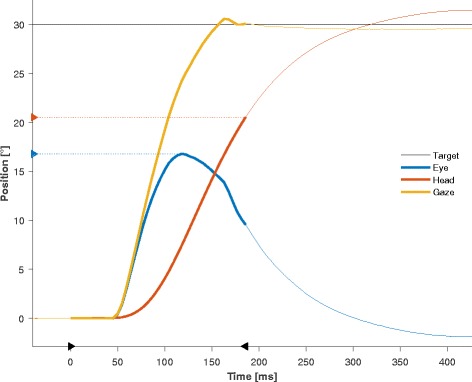
An example of the time course of a coordinated eye-head movement to a target jump of 30° consisting of a primary gaze shift (thick lines). For the primary gaze shift, it can be clearly seen that it is mainly dominated by the eye contribution during the first 50 ms. Markers on the x-axis indicate the start and end of the gaze shift. Markers on the y-axis show the peak contribution of the eye (blue) and the head (red) to the gaze shift

VOR suppression during rapid eye-head gaze shifts seems to have special characteristics. In healthy individuals, VOR suppression has been shown to antedate head motion, gaze and attention shift [13] (see also Fig. 1). Experiments analyzing the time course of VOR suppression during large eye-head gaze shifts in monkeys found maximal VOR suppression early in the gaze shift and normal VOR gain only near the end [14]. Moreover, VOR suppression seems to be a basic and task-dependent mechanism. Larger saccade amplitudes and gaze shifts are correlated with the strength of VOR suppression [15, 16]. It has been described, that position-vestibular-pause interneurons of the VOR seem to attenuate their discharge by 75 % or more during head free gaze shift in the monkey [17] but exact mechanisms between saccade generating brainstem circuits and vestibular driven eye movements are still to be investigated [15]. Again, the complex pathway of the human brain necessary to produce VOR suppression during gaze shifting also suggests a high vulnerability of this pathway to age- and disease-related brain pathology. Indeed, impaired VOR suppression has been shown to cause delayed or imprecise obstacle perception, and even relatively unspecific symptoms, such as dizziness, diplopia, blurred vision and unstableness during fast head and body movements [18–20].

### Vestibulo-ocular reflex suppression and aging

There is increasing evidence that age influences the function of VOR performance and suppression. It has been shown that older adults have a decreased ability to enhance and suppress the VOR, compared to young adults [21–23]. Moreover, several studies have shown impaired VOR suppression associated with age-related neurodegenerative diseases, like Parkinson’s disease (PD) [24–26] and progressive supranuclear palsy (PSP). PSP is a rare neurodegenerative condition with relatively rapid decline in oculomotor function, and regular occurrence of falls already in the early disease stages [27].

The positive association between intact VOR suppression during vertical smooth head movements and cognitive functioning in PSP patients might be taken as indication that both functions rely on multiple brain regions with the consequence to be similarly affected by widespread degenerative processes [28].

Recent analysis of gaze and postural coordination found age-related impairment of saccadic accuracy during postural challenge [29]. In addition, analysis of the timing of eye and body movements revealed interesting relationships. Saccade-to-stepping delays in older persons at high risk of falling may be attributed to longer central nervous system processing time necessary to plan precise foot placements [30].

### Vestibulo-ocular reflex suppression in relation to balance deficits and falls

Accumulating evidence shows a direct association between deficits in VOR suppression and falls. Eighteen older females at increased risk of falls did not suppress the VOR as effectively as 18 matched females at low risk of falling [31]. These high risk patients could not look on the floor while initiating stance from a sitting position and therefore were impaired in observing the ground surface. Di Fabio and colleagues [32] showed in a sample of 38 women with a mean age of 81.6 years that inadequate VOR suppression led to an 18-fold increased risk for falls, compared to an adequate VOR suppression.

One longitudinal study investigated oculomotor and gait and balance function using the Tinetti balance test [33]. Assessing 53 healthy older individuals annually over a time period of 9 years, the authors found a decline in VOR and VOR suppression performance correlating significantly with a decline in this test.

Stoffregen et al. found in 12 young subjects reduced postural sway variability during target oscillation compared to sway while viewing a stationary target. They stated that postural control is important for the stabilization of the visual system to allow accurate gaze movements [34]. This is interesting, as this study argues for a functional integration of postural control with visual performance. Still, analysis of simultaneously assessed VOR suppression, balance and gait parameters has not been performed yet.

Young and Hollands discovered longer saccadic reaction latencies towards a target in older participants at high risk for falls following target perturbation and required stepping adjustment [35]. Taken together, mutual influence of eye and body movements seems possible and simultaneous analysis challenging eye and body movements in a systematic approach could lead to further understanding of mechanistic aspects of the association of these systems in ageing and neurodegenerative disease.

Although it seems that the association of VOR suppression deficits and falls is mediated by postural and gait deficits, exact mechanistic data on the interaction of eye, head and body movements in older adults with and without specific balance impairments, and its association with falls, is still largely lacking.

### Current research questions and how to test them (see Fig. 2)

**Fig. 2:**
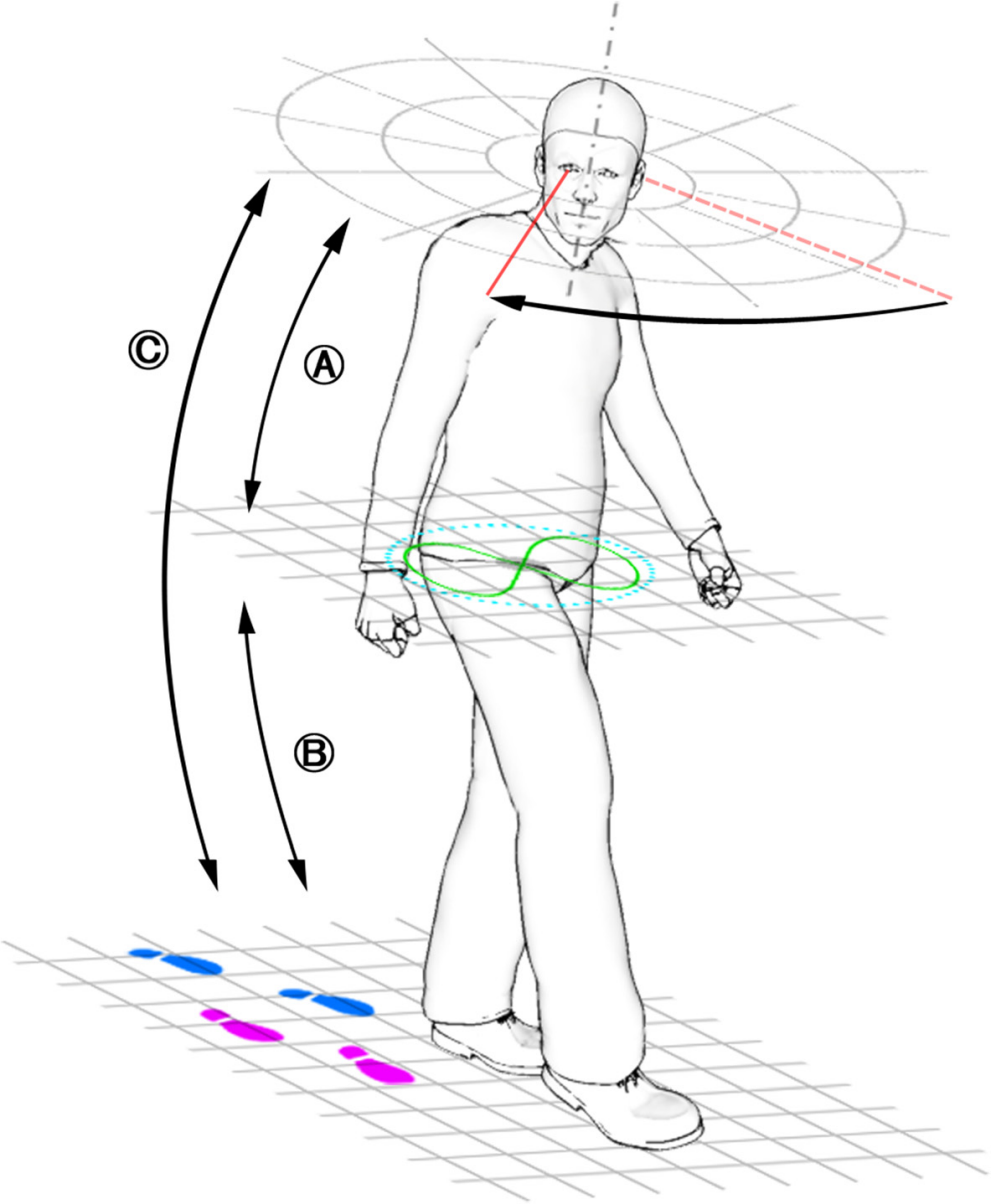
Schematic illustration of subsequent research questions, showing a subject performing a gaze shift while walking. **a** Interplay of eye/head movement and balance; **b** Interplay of balance and gait; **c** Interplay of eye/head movement and gait

(i) Is there a difference in VOR suppression ability performing slow (smooth eye/head tracking with head-fixed target) or fast head movements (rapid eye/head gaze shifts)? Combined measurement with eye/head tracker and a head-fixed target will allow analyzing VOR suppression during slow eye/head pursuit. Measurement with eye/head tracker and stimulus presentation via light emitting diodes will be used to measure the suppression ability during fast eye/head movements.
(ii) Is VOR suppression ability dependent on body position (sitting / standing / walking)? Assessment of VOR suppression ability will be performed in all three body positions.
(iii) Are VOR suppression deficits (during slow eye/head pursuit and rapid gaze shifting) associated with alterations of sway and gait parameters? Synchronized eye/head tracking and 3-D body motion analysis systems will allow the simultaneous assessment of VOR suppression ability and balance and gait parameters (see Fig. 2).
(iv) Are these gait and balance alterations able to predict falls? Prospective falls assessment will be used to analyse the validity of assessed clinical and biomechanical parameters to predict future falls.
(v) Does aging and neurodegeneration have an effect on above listed questions? Assessments will be performed in young and older healthy as well as in neurogeriatric high-risk cohorts for falls to analyse effects of aging and neurodegeneration.

### Methods/design

#### Study design

This study has a cross-sectional design to analyze the interplay between vestibulo-ocular reflex suppression, balance and gait, as well as a longitudinal design to assess their effect on future falls. All participants will obtain extensive clinical and quantitative assessment at the Robert-Bosch-Hospital Stuttgart. All procedures are approved by the local ethics committee (University of Tübingen, 602/2012BO1) and are in agreement with the Declaration of Helsinki.

### Participants

Healthy young adults and healthy older adults (age- and sex-matched to the patient cohorts) will be recruited with the support of the office of Sport and Exercise, city of Stuttgart, Germany and the Bosch BKK health insurance.

Progressive Supranuclear Palsy and Parkinson’s disease patients will be recruited by movement disorders specialists of the Center for Neurology of the University of Tuebingen. We aim at recruiting 8 infrequent fallers (≤1 fall/year) and 8 high frequent fallers (≥5 falls/ year) per cohort. Cohort size is based on feasibility (rare disease) and on comparable studies publishing significant results with similar cohort sizes (4 individuals in [4]; 8 individuals in [28]; 15 individuals in [36]).

Inclusion criteria for the patients will be probable or possible diagnosis of PSP [37] or PD (UK Brain Bank criteria [38]), disease stage according to Hoehn and Yahr stage I to IV [39], ability to walk 20 m with or without walking aid, and global cognitive test (Montreal Cognitive Assessment [MoCA] score ≥ 18).

Exclusion criteria for all cohorts will be neurological disorders (except the diagnosis of PSP or PD in the patient cohorts), manifest daily relevant dementia, psychiatric disorders, drug abuse, ophthalmologic disorders, extremity prosthesis, arthritis or musculoskeletal injuries in the past 3 months. As glasses cannot be worn using the eye-tracking device, participants requiring visual correction by glasses stronger than ±3 dpt will not be included in this study. All participants will have to give their written informed consent.

### Clinical assessment

#### Multifactorial geriatric risk assessment

Clinical data like sex, age, body mass index as well as medical history (including dose and name of drugs taken, separate listing of drugs affecting the central nervous system) will be assessed. Furthermore, comorbidity will be assessed using the *Functional Comorbidity Index* [40].

Occurrence of falls during the last week, last month, last 6 and 12 months will be inquired. For the assessment of falls-related self-efficacy, the *Short Form of the Falls Efficacy Scale-International (FES-I)* will be used. This scale has been shown to be highly related to previous and subsequent falling [41].

Activities of daily living will be assessed by the short form of the questionnaire *Late-Life Function and Disability Instrument (LLFDI)* [42].

Neuropsychological examination includes assessment of global cognition using the *Montreal Cognitive Assessment (MoCA)* [43]. Executive dysfunction will be tested using the *Trail Making Test parts A and B (TMT A + B)* [44]. The use of the *Allgemeine Depressions Skala (ADS)* [45] will help to quantify mood disturbances. Behavioral disturbance will be assessed with the *Neuropsychiatric Inventory (NPI)* [46].

To assess and compare motor impairment across different neurodegenerative diseases the *Unified Multiple System Atrophy Rating Scale (UMSARS)* will be used [47].

### Disease specific assessment

Disease severity and symptoms will be assessed by the *Progressive Supranuclear Palsy Rating Scale (PSP-RS)* [48] and the *Unified Parkinson’s Disease Rating Scale (MDS- UPDRS)* [49].

### Mobility-function assessment

For the assessment of the participants ability to adapt his gait to different tasks like head movement, turning or stair climbing the *Dynamic Gait Index (DGI)* will be used [50].

### Experimental setup (see Fig. 3)

**Fig. 3:**
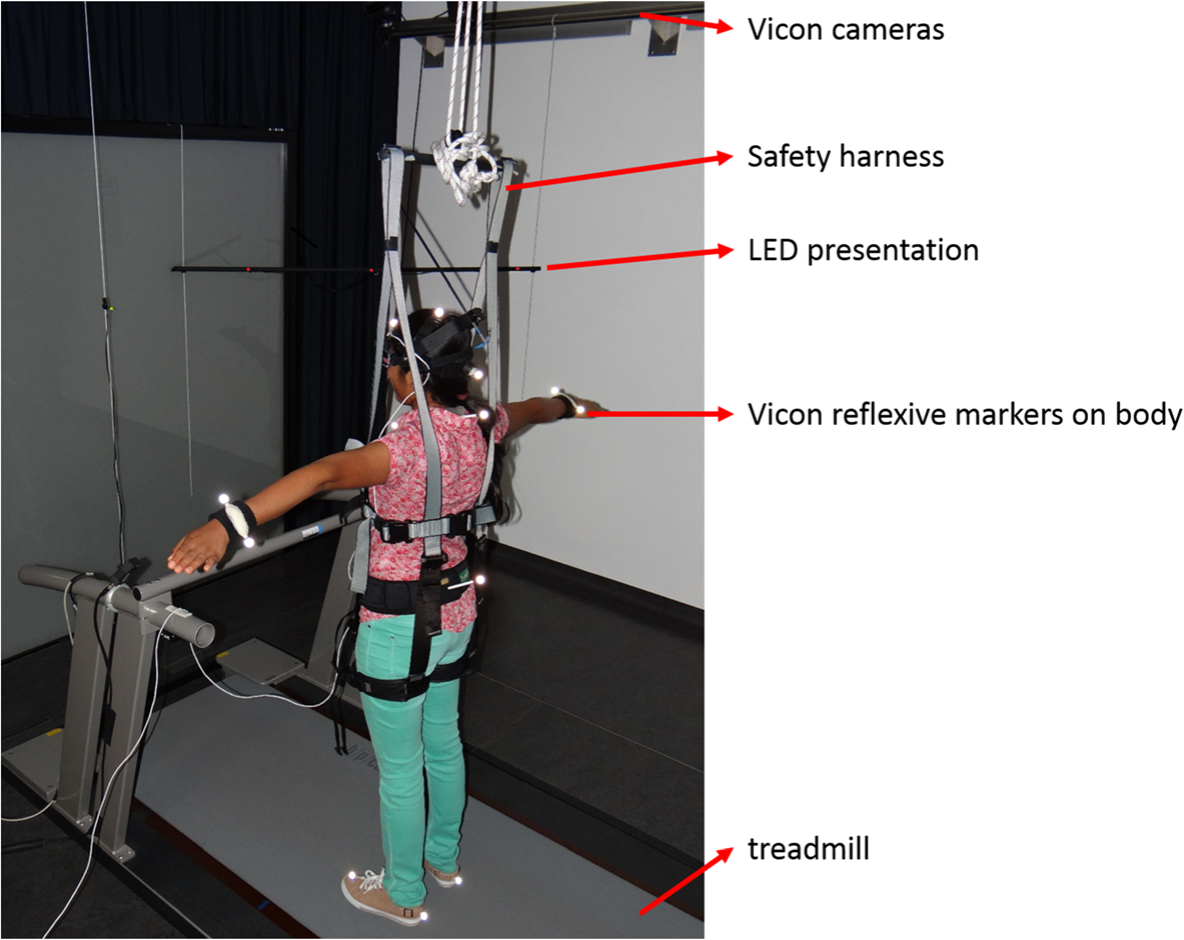
This photograph shows a doctoral student wearing a part of the experimental setup. The student is standing on the treadmill wearing a safety harness. Vicon reflective markers are placed on central positions for skeleton-fitting of the body. The central LED is positioned at 0° at a distance of 120 cm. Three LEDs are presented to the right side of the participant (left side not shown here)

#### Eye-head tracking

A mobile, video-oculography-based eye tracker (EyeSeeCam®, EyeSeeTec GmbH, Fürstenfeldbruck, Germany) will be used to record the participant’s eye and head movements. The device is head-mounted with tightly-fitting goggles and a head band to prevent movement induced slip. The eye tracker records eye-in-head movements of the left eye (sampling rate: 220 Hz, spatial resolution: 0.05 °–0.1 °, accuracy: 1 °). An accelerometer and a gyroscope record the relative head movements. Calibration is done using an integrated laser with a diffraction grating (5-point laser grid). All data is transferred to a laptop via FireWire.

### 3-D motion analysis

A six-camera VICON T10 system (Vicon^©^ Motion Systems Ltd.UK) will be used to record absolute head and body motions. The full body model consists of 15 reflective markers positioned on the head-fixed target helmet (see Fig. 3 - front, top, back), trunk (jugulum, 7^th^ cervical vertebra, 5^th^ lumbal vertebra), arms and feet.

### Stimulus presentation LEDs

Seven light emitting diodes (LEDs) as dot lights will be arranged in front of the participant (see Fig. 3). They will be positioned at 0° in front of the participant at a distance of 120 cm and at fixed positions to the left and right side of the participant. The height will be adjusted to the level of each participant’s eyes.

The LED will be controlled using a programmable microcontroller. Stimulus presentation time will be 500 ms. Stimulus presentation will be unpredictable in a random but fixed order with varying inter-stimulus-intervals to provoke fast eye-head gaze shifts.

EyeSeeCam®, the VICON system and the stimulus presentation LEDs will be synchronized via a synchronization signal.

### Body-worn sensor

To record further gait parameters we will use a wearable sensor (Dynaport Hybrid, McRoberts BV, NL) with a combined triaxial accelerometer (sampling rate 100 Hz; range ±2 g; resolution ±1 mg) and triaxial gyroscope (range ±100 °/s; resolution ±0.0069 °/s). The sensor will be worn at the lower back at the height of the second lumbar vertebra, fixed with an elastic belt.

### Treadmill

A treadmill (h/p/cosmos venus; length 200 cm, width 75 cm, h/p/cosmos sports medical GmbH, Germany) will be used for the walking paradigm. Participants will be allowed to get accustomed to treadmill walking before the trial start. Comfortable gait speed and fast gait speed will then be estimated and documented in m/s.

### Experimental protocol (see Table 1)

**Table 1:** Experimental design to allow simultaneous analysis of vestibulo-ocular reflex, vestibulo-ocular reflex suppression and balance (while standing) and balance and gait (while walking)

	Sitting	Standing	Walking
Head-impulse test (VOR) passive	✓		
Head-impulse test (VOR) active	✓	✓	✓
Smooth eye-head tracking passive	✓		
Smooth eye-head tracking active	✓	✓	✓
“Natural” walking			✓
Fixation-/no-fixation		✓	✓
Rapid eye-head gaze shifts	✓	✓	✓

Measurements will be performed in three different conditions: Sitting, Standing, and Walking. Passive = head movement made by examiner. Active = head movement made by subject (important while standing and walking, as the examiner should not influence the subjects balance and gait). VOR = vestibulo-ocular reflex. Smooth eye-head tracking = vestibulo-ocular reflex suppression test with head-fixed target. “natural” walking = treadmill walking without presentation of visual stimuli (control task). Fixation-/no-fixation = standing or walking 30 s. without fixation of a visual target, standing or walking 30 s. while fixation of a visual target. Rapid eye-head gaze shifts = vestibulo-ocular reflex suppression test while rapid gaze shifts

Prior to each condition and in case of slipping of the eye-tracking system, calibration will be performed.

Subjects will be asked to reduce blinking if possible to avoid data loss in the eye position. Artificial tears will be provided, if necessary.

In all procedures data quality will be visually monitored in real time and additional trials will be completed when needed, to ensure enough trials with good quality for analysis.

### Vestibulo-ocular reflex (Head-impulse test, HIT)

VOR function will be tested first in the sitting condition (see Additional file 1: Video A). First the response to passive yaw head impulses will be investigated [51], which is an unpredictable horizontal head rotation of low amplitude, high acceleration and high peak velocity.

The examiner will be standing behind the participant, asking him to relax the shoulders and neck muscles. The participant will be required to continuously fixate the earth-fixed central LED target during the impulses.

### Vestibulo-ocular reflex suppression with head-fixed target (VOR S)

**Fig. 4:**
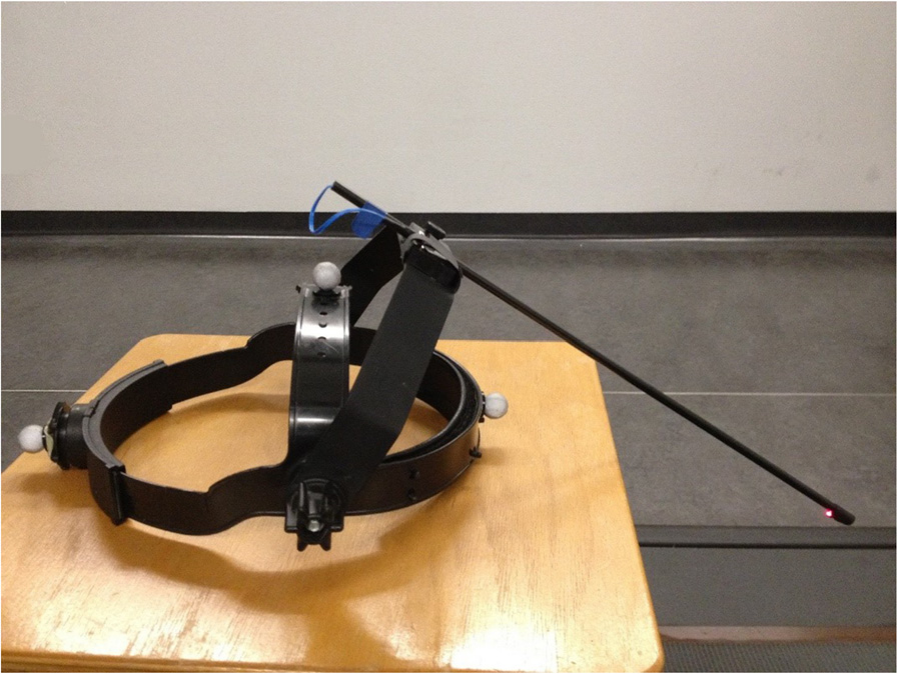
Custom made head-fixed target with reflective Vicon marker

VOR suppression during smooth eye-head tracking will be assessed using a head-fixed target (see Fig. 4). The fixation LED will be positioned 30 cm in front of the participant’s eyes. First, VOR suppression will be tested passively with head movements performed by the examiner standing behind the participant. The participants head will be rotated at approximately 0.2 Hz. The participant will be instructed to continuously fixate the head-fixed target positioned in front of his eyes. The participant’s oculomotor performance can be continuously controlled by the examiner by real-time signal observation on the laptop screen.

After the passive VOR suppression performance, active VOR suppression performance (head movement without control by the examiner while fixation of the head-fixed target) will be assessed (see Additional file 2: Video B). The participant will be allowed to practice the active VOR suppression performance to adapt velocity and amplitude to the passive VOR suppression performance. Passive and active VOR suppression performance will be assessed in the horizontal and vertical plane.

This VOR suppression testing will also be performed in the standing and walking condition. Safety will be ensured by the use of a relatively unobtrusive harness. During the standing trials, the participant will be asked to stand relaxed with his hands by his side while performing sinusoidal head movements and fixating the head-fixed LED target. The active VOR suppression will be performed at steady-state gait speed during walking on the treadmill at the previously selected individual comfortable gait speed (see Additional file 3: Video C).

### Rapid eye-head gaze shifts

Gaze shifting ability will be tested first in the sitting condition (see Additional file 4: Video D).

The participant will be asked to fixate the central LED (0 °). The order of conditions will be fixed to sitting (1.), standing (2.) and walking (3.), as counter-balancing does not seem reasonable due to the limited group size. The participant will be asked to fixate the LEDs “as fast as possible” whenever they appear and after each fixation, re-fixate the central LED.

Safety will again be ensured by the use of the harness. During the standing trials, the participants will be asked to stand relaxed with their hands by their side facing the central LED. Then the gaze shifting will be performed at steady-state gait speed during walking on the treadmill at the previously selected individual comfortable gait speed (see Additional file 5: Video E).

### Experimental variables

#### Eye and head movements

Raw data will be processed offline using MATLAB (Matlab R2013b, MathWorks, Natick, MA) software.

Eye position, eye velocity and head velocity will be taken from the values returned by the eye tracker software, which applies an internal blink-removal method and a Gaussian zero-phase low-pass filter to the eye data. All data will be upsampled using a piece-wise cubic Hermite polynomial interpolation to match the sampling rate of the Vicon data (1 kHz). Eye and head accelerations will be computed from head velocity with a central difference method. Head position will be acquired from head velocity using a detrended cumulative sum.

##### HIT detection

Saccades and head impulses will be automatically detected using a two-stage head velocity threshold and a 20 ms moving standard deviation for eye acceleration. All detected head impulses will be visually inspected and corrected if needed. Valid trials will be determined based on peak head velocity and acceleration as well as eye velocity smoothness, e.g., the standard deviation of the difference of successive velocity samples. Trials in which the overall smoothness exceeds 30°/s will be excluded, since the pupil is not correctly detected by the eye tracker. At the beginning of each trial, maximum head velocity will have to be lower than 40 °/s. For a trial to be valid, peak head velocity will have to be between 45°/s and 500 °/s and will have to occur within 60 and 200 ms after onset of the head movement. Peak head acceleration will have to lie between 1.000 °/s^2^ and 10.000 °/s^2^. Trials, in which an eye movement of more than 1° in the same direction as the head movement occurs, will be excluded as anticipatory response.

Over all trials, the following parameters will be determined: Median instantaneous velocity gain (−eye velocity / head velocity) at 40, 60 and 80 ms after head impulse onset and mean peak head velocity, acceleration and deceleration with corresponding times from onset. To get a measure for the symmetry of the head movement, the signed acceleration amplitude (absolute peak deceleration - absolute peak acceleration) will be computed, which yields values close to 0 for very symmetric movements, but normally is negative since accelerations tend to be higher than decelerations. Head-impulse velocity gain of the VOR will be calculated with a robust ordinary least-squares regression from eye and head velocity [52]. All output parameters will be generated for the left and right side separately.

##### VOR suppression (VORS) during smooth eye-head tracking with head-fixed target

A head velocity threshold of 10 °/s will be used for the detection of the VORS period. Saccades will be detected with a 15 ms moving standard deviation of the eye position. Saccades with amplitudes smaller than 1°, durations less than 5 ms and peak velocities outside the range of 20 to 1000 °/s will be considered artefacts and removed. For the horizontal and vertical dimension, saccades will be grouped in the corresponding hemifield (left/right, up/down) and their number, frequency, mean amplitude, duration and peak velocity are computed. VORS quantity will be assessed for each direction with the median position gain (eye position / head position), standard deviation of the eye position and the saccade frequency over the whole period.

In addition, peak head frequency (*f*_max_^*H*^) and the corresponding amplitude (*a*_max_^*H*^) will be derived from the Fourier transform of the head velocity. The amplitude in the Fourier transform of the eye velocity at the peak head frequency (*a*^*E*^) will be computed and a compensation factor for the VORS will be derived from *C* = (1 − *a*^*E*^/*a*_max_^*H*^) ⋅ 100. The compensation factor ranges from 0 % at no VORS to 100 % at perfect VORS.

##### Rapid eye-head gaze shift

Gaze position will be computed through linear addition of eye and head position. The target appearance will be externally controlled and the control signal will be send to the Vicon software. To synchronize this signal with the gaze data, participants will have to move their head up and down in the beginning of the gaze shift experiment to get a strong peak in head position in both datasets. The lag between eye tracker and Vicon data will be accordingly corrected using a cross-correlation procedure. Saccades will be detected similarly as in the VORS trials but with a larger window size of 150 ms and only saccades with amplitudes of more than 5 ° and durations longer than 25 ms will be considered. For the first detected saccade in each trial that occurred within 2 s of the target onset, reaction time, robust start and end positions (median position over 100 ms before/after the on-/offset), robust amplitude (robust end position - robust start position) and duration will be computed. To measure the accuracy of the gaze shift, the robust position gain (robust end position / target position) will be determined. To quantify the dynamic properties of the saccades, the main sequence will be computed for duration versus robust amplitude with a robust linear least-squares regression [52].

#### Body movements

The gait parameters will be selected on previously established associations with fall risk [53, 54].

##### 3-D Motion capture system (Vicon)

Mediolateral displacements of body segments [55] as well as the gait phases and their durations will be calculated. The analysis of dynamic stability at touchdown of the right and left foot will be aimed at using the margin of stability. It is determined as the difference between base of support and extrapolated center of mass (CoM) [56]. The extrapolated CoM can be defined by calculation of the horizontal component of the projection of the CoM to the ground, the horizontal CoM velocity and the distance between CoM and center of ankle joint [57].

Furthermore, for the analysis of the coordination of body segments, maximum segment rotation, maximum angular velocity, range of the angular velocity and parameters of timing will be calculated. Similar measures have previously been published [58, 59].

##### Dynaport Hybrid

Sway parameters, such as mediolateral and anteroposterior postural sway length and velocity will be calculated for analysis of postural stability during static standing [60, 61].

Parameters related to dynamic stability in walking including stride-time, stride-time variability and stability of gait (LDS) will be generated [62, 63].

### Prospective falls assessment

All participants will receive a prospective fall protocol (expert consensus; http://farseeingresearch.eu). They will be asked to document any fall during the next year. A study nurse will remind the participants once a month via telephone call to fill in the protocol, and monitor the fall protocols completed for each fall so far. After 1 year, the fall protocol of each subject will be collected.

## Discussion

Reduction of falls frequency in an ageing society is a major aim of health care. Exact mechanisms of suspected causal factors like VOR suppression deficits are yet unknown and a systematic analysis of these mechanisms is urgently needed. This study protocol describes an experimental setup allowing the analysis of simultaneously assessed eye, head and body movements. It helps to further understand the association of VOR suppression deficit and falls. Prospective falls assessment within this study will eventually enable a calculation of the predictive validity of the clinical and biomechanical variables.

### Study strengths and limitations

To the best of our knowledge, this is the first study to assess high-frequency VOR, VOR suppression, balance and gait simultaneously under quantitative experimental conditions.

Further, it includes neurogeriatric cohorts at high risk for falls. Patients will be selected by movement disorders specialists at the Department of Neurodegeneration, Center for Neurology, University Hospital Tuebingen.

Nevertheless, our study has some limitations. First, the sample size is relatively small. However, participants will be tested extensively in a very standardized protocol. This setting will allow the extraction of relevant information for future, more hypothesis-driven studies.

Although our setup approaches natural conditions by extending the examination of VOR-suppression to standing and walking, it remains artificial insofar that subjects walk on a treadmill in a sparse and dark environment. In principle, translation of our results into real-life outdoor conditions might be considered in future experiments. However, we believe that this setup allows precise and detailed analysis of the association of VOR suppression deficits and balance and gait measures and leads to a better understanding of the interaction between the oculomotor and the locomotor systems.

## Conclusions

Our protocol uniquely combines sound methods in analyzing mechanistic aspects of the association of oculo- and locomotion and a clinically relevant prospective falls assessment in ageing and neurodegenerative disease. We will investigate established and novel markers of fall risk related to VOR suppression deficit, gait and balance using a new setup with high technical standard. The results will help to better understand the mechanisms of falls and to design new therapeutic interventions.

## Additional files

Additional file 1: Video A. (MP4 12373 kb) Additional file 2: Video B. (MP4 13973 kb) Additional file 3: Video C. (MP4 11979 kb) Additional file 4: Video D. (MP4 5153 kb) Additional file 5: Video E. (MP4 18703 kb)

## Abbreviations

ADS: Allgemeine Depressions Skala
CoM: Extrapolated center of mass
DGI: Dynamic Gait Index
FES-I: Short Form of the Falls Efficacy Scale-International
HIT: Head-impulse test
LLFDI: Late-Life Function and Disability Instrument
LDS: Local dynamic stability
LED: Light-emitting diodes
MoCA: Montreal Cognitive Assessment
NPI: Neuropsychiatric Inventory
PSP: Progressive Supranuclear Palsy
PSP-RS: Progressive Supranuclear Palsy Rating Scale
PD: Parkinson’s disease
TMT A + B: Trail Making Test parts A and B
UMSARS: Unified Multiple System Atrophy Rating Scale
MDS-UPDRS: Unified Parkinson’s Disease Rating Scale, Movement Disorders Society
VOR: Vestibulo-ocular reflex
VOR S: The suppression of the vestibulo-ocular reflex
VOR gain: Eye velocity/head velocity
VICON: 3-D motion capture system
